# Decreased Functional Diversity and Biological Pest Control in Conventional Compared to Organic Crop Fields

**DOI:** 10.1371/journal.pone.0019502

**Published:** 2011-05-18

**Authors:** Jochen Krauss, Iris Gallenberger, Ingolf Steffan-Dewenter

**Affiliations:** 1 Department of Animal Ecology and Tropical Biology, University of Würzburg, Biocentre, Würzburg, Germany; 2 Population Ecology Group, Department of Animal Ecology I, University of Bayreuth, Bayreuth, Germany; University of Zurich, Switzerland

## Abstract

Organic farming is one of the most successful agri-environmental schemes, as humans benefit from high quality food, farmers from higher prices for their products and it often successfully protects biodiversity. However there is little knowledge if organic farming also increases ecosystem services like pest control. We assessed 30 triticale fields (15 organic vs. 15 conventional) and recorded vascular plants, pollinators, aphids and their predators. Further, five conventional fields which were treated with insecticides were compared with 10 non-treated conventional fields. Organic fields had five times higher plant species richness and about twenty times higher pollinator species richness compared to conventional fields. Abundance of pollinators was even more than one-hundred times higher on organic fields. In contrast, the abundance of cereal aphids was five times lower in organic fields, while predator abundances were three times higher and predator-prey ratios twenty times higher in organic fields, indicating a significantly higher potential for biological pest control in organic fields. Insecticide treatment in conventional fields had only a short-term effect on aphid densities while later in the season aphid abundances were even higher and predator abundances lower in treated compared to untreated conventional fields. Our data indicate that insecticide treatment kept aphid predators at low abundances throughout the season, thereby significantly reducing top-down control of aphid populations. Plant and pollinator species richness as well as predator abundances and predator-prey ratios were higher at field edges compared to field centres, highlighting the importance of field edges for ecosystem services. In conclusion organic farming increases biodiversity, including important functional groups like plants, pollinators and predators which enhance natural pest control. Preventative insecticide application in conventional fields has only short-term effects on aphid densities but long-term negative effects on biological pest control. Therefore conventional farmers should restrict insecticide applications to situations where thresholds for pest densities are reached.

## Introduction

Ecosystem services like pollination and pest control are essential benefits for farmers throughout the world [1]–[3]. Pollinators enhance crop production for many cash crops like fruits and vegetables [4] and biological pest control is an important ecosystem service for crops [5], [6]. In the last century agricultural intensification caused significant biodiversity loss in most agroecosystems, underlying the need for restoration and conservation schemes in agroecosystems [5], [7]. Biodiversity and ecosystem services might be protected with agri-environmental schemes, where farmers get subsidies, partly to produce ecological benefits. Some of these schemes have been criticised, due to their low success in protecting biodiversity [8], while other schemes were successful [9], [10].

One important agri-environmental scheme is organic farming, where synthetic fertilisation and pesticide treatments are not applied, while both are common in conventional farming systems. Organic farming might decrease the biomass of the crop by 25% [11], but increases the diversity of most functional species groups [6], [12]–[18], but see [19] for an exception. Particularly, organic farming enhances guilds relevant for ecosystem services like pollinators [20] and predators [21]. Studies focusing in parallel on species diversity of different functional groups and ecosystem services are rare. In a recent review on pest control in organic and conventional farms, the authors call for additional studies on the relationship of biodiversity and pest control [6]. In this context, field edges and field centres often contain different species communities, with higher diversities, abundances and ecosystem service provision at the edges compared to centres [5], [18]. It is therefore necessary to consider in field studies edges and centres separately.

Aphids are major insect pests on cereals and can cause massive yield loss [22], [23]. An application of systemic insecticides in conventional fields is therefore a common practice [24]–[26]. Unexpectedly, the application of insecticides was not a common praxis in our study region. Therefore we had the chance to compare conventional fields with and without insecticide application. As aphid predators might be similarly reduced by insecticides as aphids, and as aphid population growth rates are very high [27], [28], it is plausible that top-down control could be reduced in fields with insecticide treatment [26].

Most studies comparing cereals of organic vs. conventional farming systems were conducted in wheat fields [13], [14], [20] with some studies on barley and oat fields [29]. Beside the field scale some studies focused on field margins [30], on effects of organic farming at the landscape scale [31] or use a farm scale approach [32]. As far as we know, studies on triticale were not performed at any spatial scale. Triticale is a cereal emerged from crossing and backcrossing of wheat (*Triticum aestivum* L.) and rye (*Secale cereale* L.) and is mainly used in low-input systems as animal feed. Its importance might grow because of its potential role in biofuel production [33], [34]. The worldwide production in the year 2009 was 15,040,432 t and therefore comparable with rye 17,856,568 t and oat 23,032,118 t, but far below maize 817,110,509 and wheat 681,915,838 (FAOSTAT 2009: http://faostat.fao.org/).

In this study we compared conventional and organic fields of the cereal triticale, distinguishing between effects of field edges and centre of the fields, and considering the diversity of functional groups including plants and pollinators as well as densities of aphids and predators, and predator-prey ratios. We further tested the effect of insecticide treatment on aphids and their predators in conventional fields.

We tested the following hypotheses:

1. Vascular plant and pollinator diversity is enhanced in organic compared to conventional farming.
2. Higher predator abundance (pest control) leads to reduced aphid abundance in organic compared to conventional farming systems.
3. Field edges are more species rich and contain higher abundances than field centres.
4. In conventional fields sprayed with insecticides herbivore abundances recover faster than predator abundances.

## Materials and Methods

### Study region and study sites

**Figure 1:**
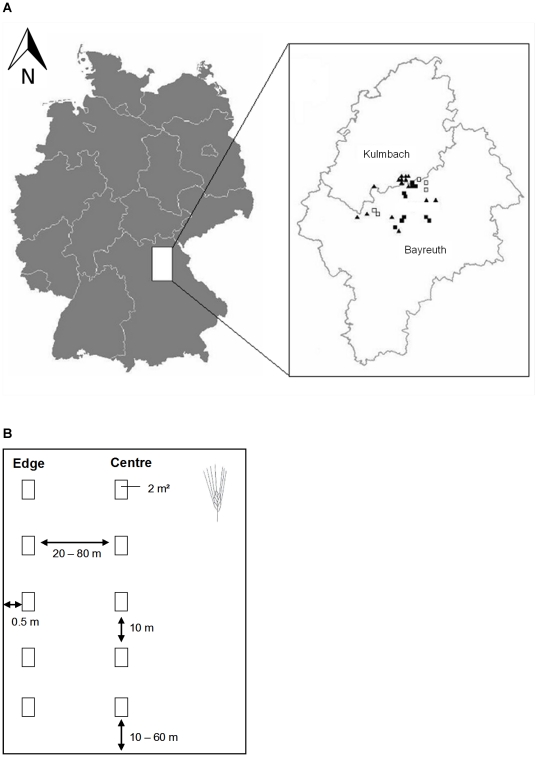
Location of study region in southern Germany and used sampling scheme. (a) The 30 triticale fields were located in the northern part of Bavaria in the districts of Bayreuth and Kulmbach (Symbols: ▪ conventional, not treated; □ conventional treated with insecticides; ▴ organic farming system). (b) Sampling scheme in each triticale field. Five study plots were surveyed at the edge and five in the centre of each field.

A total of 30 (15 organic, 15 conventional) winter triticale fields were selected as study sites in the vicinity of Bayreuth (49°56′53″N, 11°34′42″E) located in the region Upper Franconia (South Germany, Bavaria) (Fig. 1a). The study sites were within an area of approximately 300 km^2^ and the minimum distance between the studied triticale fields was 500 m. Upper Franconia is characterised by relatively heterogeneous landscapes, rich in forests (40.4% of the land area) and agricultural land (47.3%) including arable land (69.1%), grassland (30.5%) and permanent crops (0.4%) (Bayerisches Landesamt für Statistik und Datenverarbeitung 2004; http://www.statistik.bayern.de). The average temperature in the study region (Bayreuth: 1971–2000) is approximately 8.2°C with an average rainfall of 724 mm per year (http://www.klimadiagramme.de/Deutschland).

The 15 conventional fields, we investigated, were treated with agrochemicals like herbicides, inorganic fertilisers and growth regulators (for detailed information see Table 1). For cereal aphid control five of the 15 conventional fields were sprayed preventatively with the insecticides Karate®Zeon (Syngenta) with 75 ml/ha and Pirimor (Syngenta) with 100 g/ha. Karate®Zeon is a contact insecticide against sucking and chewing herbivores and contains lambda-cyhalothrin, a pyrethroid, as active agent. Pirimor comprises the cabarmate Primicarb and, similar to Karate®Zeon influences the nervous system and leads to paralysis and mortality, but Pirimor is specific against aphids [35] and (Syngenta product information http://www.syngenta.de/).

**Table 1:** Application of agrochemicals on conventional triticale fields in the region of Oberfranken (x = treated).

Field number	Field name (location)	Inorganic fertiliser	Herbicide	Insecticide	Fungicide	Growth regulator
1	Eichelberg 1	X	X		X	
2	Eichelberg 2	X	X		X	
3	Geigenreuth 1	X	X			
4	Geigenreuth 2	X	X			
5	Mistelbach	X	X		X	
6	Obergräfenthal 1	X	X			X
7	Obergräfenthal 2	X	X			X
8	Obergräfenthal 3	X				
9	Unterkonnersreuth 1	X	X			X
10	Unterkonnersreuth 2	X	X			
11	Crottendorf	X	X	X	X	
12	Eschen	X	X	X	X	
13	Ramsenthal	X	X	X	X	
14	Schaitz	X	X	X	X	
15	Windhof	X	X	X	X	

By contrast the 15 organic fields were cultivated under the European Union regulation (EEC) N° 2092/91 based on a prohibition of inorganic fertilisers and pesticide application.

Forest directly adjacent to the 30 study fields was recorded to test if forest act as a source habitat for species [36], [37]. However, the proportion of forest surrounding the fields had no significant effect on species richness and abundance (all p>0.2) and was therefore excluded from all statistical models and is not presented in the results.

### Data collection

#### Study design

We established 10 study plots on each of the 30 triticale fields. Five plots were located at the edge (0.5 m away from the outer field border) and 5 plots in the centre of the fields (Fig. 1b). The study plots had an area of 2 m^2^ and were arranged in a row along the edge or centre every 10 m. Depending on the field size there was a distance of 20 to 80 m between the study plots at the edge and those in the centre of the fields (Fig. 1b).

#### Vascular plants and pollinators

Vascular plant species richness was recorded once in a random sequence between the 5^th^ and the 27^th^ June 2008 in the 10 plots on all 30 triticale fields. Most arable wild plants were determined directly in the fields; unknown species were taken into laboratory for subsequent identification. The vegetation cover was estimated by vertical projection of non crop plant elements on the ground. For statistical analyses we used the total number of plant species at the edge and in the centre of the field, while the vegetation cover is the mean of the 5 plots (field edge or field centre).

Pollinators were recorded between 10^th^ of June and the18^th^ of July 2008 in 50 m×2 m transect corridors, separately conducted for field edges and field centres. The walks were repeated three times at different days and lasted approximately 10 minutes for each transect corridor. All flower-visiting insects of the families Apidae, Syrphidae and Lepidoptera were recorded. Unknown species were netted and taken into laboratory for subsequent identification. For statistical analyses we used the total number of species and the summed number of individuals of the three walks to calculate abundances (separately for field edges and field centres).

#### Cereal aphids and their natural enemies

In 2008 cereal aphids and their natural enemies were recorded in four surveys at different days in the period between the 10^th^ of June and the 19^th^ of July. On each of the 10 study plots per triticale field (5 edge, 5 centre) 10 sweeps with a net were carried out to count cereal aphids and aphidophagous predators in the nets. We focused our study on three cereal aphid species which are known as pests in European agroecosystems: *Sitobion avenae* (Linnaeus), *Metopolophium dirhodum* (Walker) and *Rhopalosiphum padi* (Fabricius) (Hemiptera, Aphididae) [21], [23]. We also recorded specialised aphidophagous predators, including all larvae and adults of the ladybirds *Coccinella septempunctata* (L.) and *Prophylea quatuordecimpunctata* (L.) (Coccinelidae, Coleoptera), lacewing larvae (Chrysopidae) and hoverfly larvae (Syrphidae). These stenophagous predators are known to contribute effectively to cereal aphid control [23], [38]. For statistical analyses we used the summed individual numbers of the four surveys, whereby each survey contains the sum of the five study plots (either at the field edge or the field centres). The predator-prey ratio was calculated by dividing the number of (aphidophagous) predators by the number of prey (cereal aphids). To assess the temporal dynamics of aphids and their predators on conventional fields with and without insecticide application we also tested aphid and predator abundances for each survey separately. Temporal dynamics were not considered for pollinators, as they occurred in too low densities throughout the season.

### Statistical analyses

The statistical analyses were performed using the software R 2.9.1 for Windows [39]. Linear mixed effects models were calculated with the sequence of explanatory variables being (i) farming system (conventional/organic), (ii) field position (edge/centre), and (iii) the interaction between farming system and field position. Edge and centre plots or transects were nested within fields by using field identity as random effect [40]. The four time steps in the insecticide treatment analyses were performed separately apart from one comparison between time step 1 and time step 4 in aphid abundances. In this specific case we added time step as fixed effect with explanatory variable interactions and further nested it as random effect within field identity. The response variables plant species richness, pollinator species richness, pollinator abundance and predator abundance were log_10_ (c+0.1) and aphid individuals log_10_ - transformed to meet the assumptions of normality and homoscedasticity in the statistical models. Vegetation cover and predator-prey ratios were not transformed. Pearson correlations were used to show relations between response variables (Table 2). Means ± one standard error are presented throughout the text.

**Table 2:** Pearson correlations (r-values) between response variables of the 60 study locations in 30 fields.

	Vegetation cover	Pollinator species richness	Pollinator abundance	Aphid abundance	Predator abundance	Predator-prey ratio
Plant species richness	0.72****	0.73****	0.77****	−0.52****	0.60****	0.53****
Vegetation cover		0.77****	0.83****	−0.65****	0.56****	0.79****
Pollinator species richness			0.98****	−0.75****	0.49***	0.60****
Pollinator abundance				−0.75****	0.52****	0.66****
Aphid abundance					−0.21 ns	−0.56****
Predator abundance						0.59****

Variables are transformed (see statistical analyses).

Significance levels:

****P<0.0001;

***P<0.001;

n.s. = not significant.

## Results

### Vascular plants and pollinators

**Figure 2:**
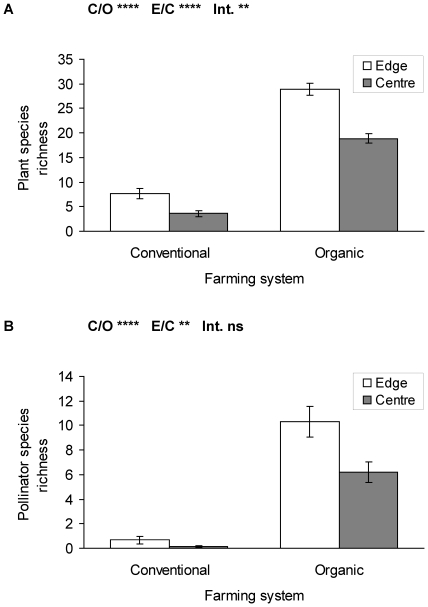
Species richness of (a) vascular plants and (b) pollinators in conventional and organic triticale field edges and centres (mean ± se). C/O: conventional/organic fields, E/C: edge/centre in field, Int: Interaction term. **** p≤0.0001, ** p≤0.01, ns p>0.1. Statistics see Table 3.

In the vascular plant surveys 55 weed species were found in conventional and 114 species in organic fields (in total 122 species, see Material S1, Appendix A). The species richness and the vegetation cover of non-crop species were significantly higher in organic fields compared to conventional fields. Further, field edges showed consistently higher species richness and vegetation cover compared to field centres (Fig. 2a; Table 3; vegetation cover: conventional/edge = 2.4±0.7%, conventional/centre = 1.1±0.6%; organic/edge = 30.7±2.4%; organic/centre = 16.3±2.3%). The interaction terms between the explanatory variables was also significant, indicating that the difference between edges and centres is more pronounced in organic fields.

**Table 3:** Mixed effects model statistics of the seven response variables with the explanatory variables farming system, field location and the interaction of farming system and field location.

	df		Plant species richness	Vegetation cover	Pollinator species richness	Pollinator abundance	Aphid abundance	Predator abundance	Predator-prey ratio
Farming system (conventional/organic)	1,28	*F*	96.99	86.01	134.32	180.16	91.04	12.90	25.60
		*P*	<0.0001	<0.0001	<0.0001	<0.0001	<0.0001	0.001	<0.0001
			↓	↓	↓	↓	↑	↓	↓
Field location (edge/centre)	1,28	*F*	71.13	66.27	10.88	35.45	13.35	26.04	11.33
		*P*	<0.0001	<0.0001	0.003	<0.0001	0.001	<0.0001	0.002
			↑	↑	↑	↑	↑	↑	↑
Framing system×Field location	1,28	*F*	7.89	46.44	<0.01	5.13	7.63	0.44	9.91
		*P*	0.009	<0.0001	0.971	0.032	0.010	0.515	0.004

**Figure 3:**
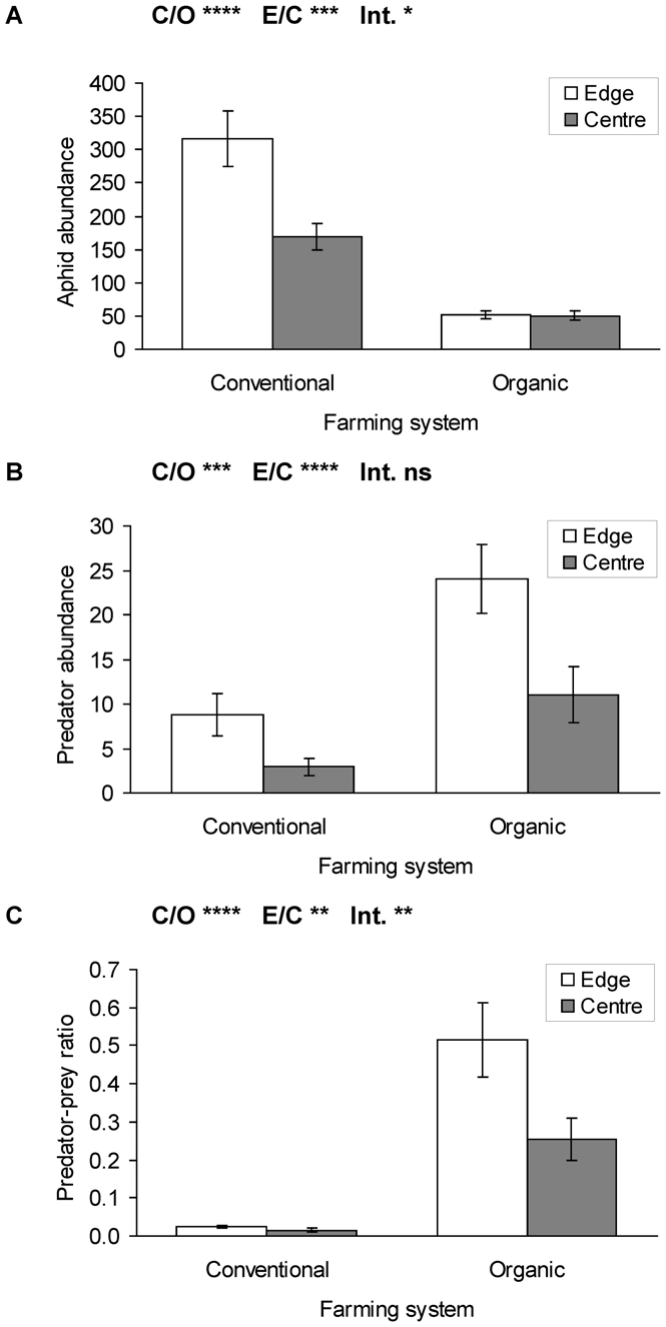
Abundances of (a) aphids, (b) aphid-predators and (c) predator-prey ratio in conventional and organic triticale field edges and centres (mean ± se). C/O: conventional/organic fields, E/C: edge/centre in field, Int: Interaction term. **** p≤0.0001, *** p≤0.001, ** p≤0.01, * p≤0.05, ns p>0.1. Statistics see Table 3.

Field identity was used as a random effect. Response variables were transformed (see statistical analyses). Mean and SE are shown in Fig. 2 and Fig. 3 or in the text.

↑ = conventional or edge is higher, ↓ = conventional or edge is lower.

In total 31 species and 3113 individuals of potential pollinators were recorded (Material S1, Appendix B). Species richness and abundance of pollinators showed consistently similar patterns as vascular plants because they were highly correlated (Table 2). Pollinator species richness and abundance were significantly higher in organic compared to conventional fields, and higher at edges compared to field centres (Fig. 2b; Table 3; pollinator abundance: conventional/edge = 1.5±0.8 individuals, conventional/centre = 0.1±0.1 individuals; organic/edge = 167.6±45.7 individuals; organic/centre = 38.3±9.8 individuals). Thereby pollinator species richness with a total of only 5 recorded species in all 15 conventional fields was substantially lower than the 31 species found in the 15 organic fields.

The interaction term (conventional/organic vs. edge/centre) was not significant for pollinator species richness, but was just below the significance level for pollinator abundance (Table 3), indicating that abundance differences in organic fields between edge and centre were slightly more pronounced than for species richness. The edges to centre differences for pollinators are very small in conventional fields, due to low individual numbers at both field locations.

### Cereal aphids and their natural enemies

A total of 8835 aphid individuals were collected in the 30 triticale fields. Altogether, almost five times more aphids were recorded in the 15 conventional fields (7296) than on the 15 organic fields (1539). *Sitobion avenae* (Fabricius) was the most dominant species (90%), followed by *Rhopalosiphum padi* (L.) (5.4%) and *Metopolophium dirhodum* (Walker) (4.6%). Aphid abundance was significantly higher in conventional compared to organic fields (Fig. 3a, Table 3), contrasting the patterns of pollinator and vascular plant abundance, which were lower in conventional compared to organic fields. Field edges in conventional fields contained much higher aphid abundances than field centres, whereas the low aphid numbers in organic fields did not differ between edges and centres (Fig. 3a, Table 3).

The abundance of aphid predators, in contrast to their prey, was higher in organic compared to conventional fields. Further, the field edges had consistently higher abundances than field centres. The interaction was not significant (Fig. 3b, Table 3), indicating that organic and conventional fields have similarly two to three times higher predator abundances at field edges compared to field centres. Due to the higher predator, but lower aphid abundances in organic fields, the predator-prey ratio in organic fields was 21 times higher at field edges and 16 times higher in field centres compared to conventional fields (Fig. 3c, Table 3).

### Insecticide treatment on conventional farms

**Figure 4:**
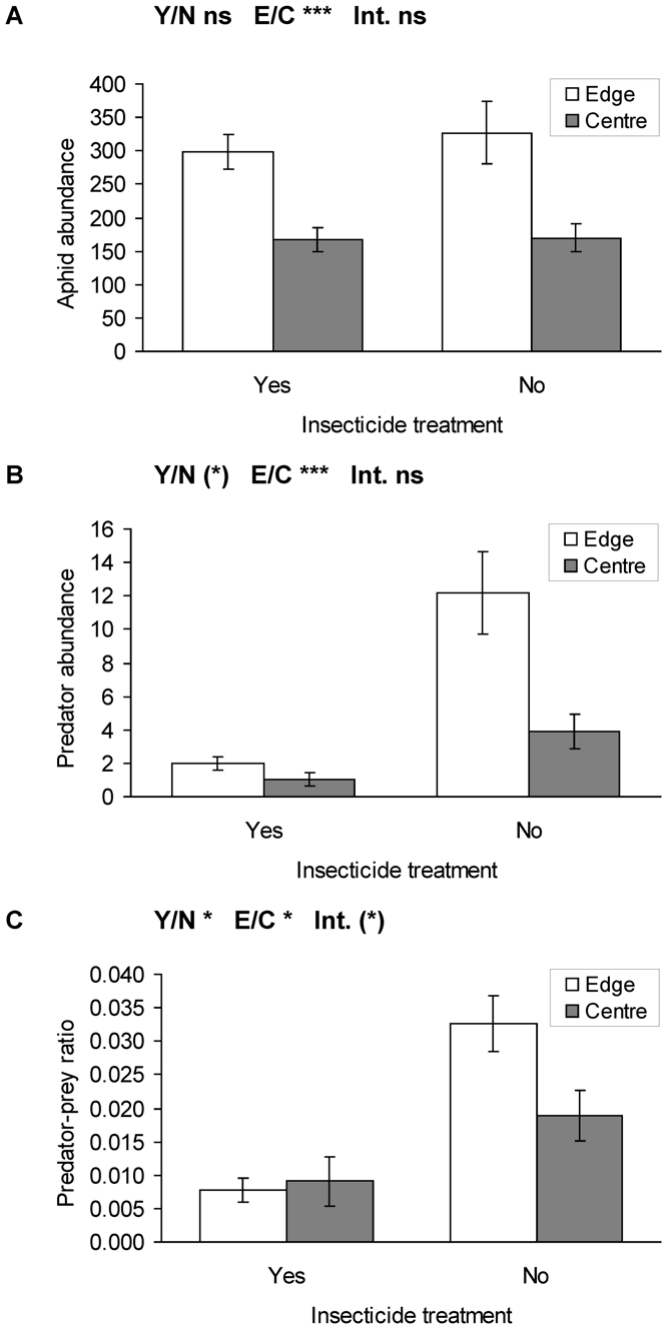
Abundances of (a) aphids, (b) aphid-predators and (c) predator-prey ratio in insecticide treated and non-treated conventional triticale field edges and centres (mean ± se). Y/N: treated/not treated fields, E/C: edge/centre in field, Int: Interaction term. *** p≤0.001, * p≤0.05, (*) p≤0.1, ns p>0.1. Statistics see Table 4.

Five of the 15 conventional field sites were treated with insecticides before the surveys started. However the aphid abundances were not significantly different between sprayed and unsprayed field sites, while higher abundances were detected at field edges compared to field centres (Fig. 4a, Table 4). In contrast, the aphid predators were more abundant in not sprayed fields, being also higher at edges compared to field centres (Fig. 4b, Table 4). Thus, the predator-prey ratio was significantly higher in not sprayed conventional fields compared to insecticide treated fields (Fig. 4c, Table 4).

**Table 4:** Mixed effects model statistics for aphid abundance, predator abundance and predator-prey ratio with the explanatory variables insecticide treatment, field location and the interaction of insecticide treatment and field location.

	df		Aphid abundance	Predator abundance	Predator-prey ratio
Insecticide treatment (yes/no)	1,13	*F*	<0.01	4.58	5.16
		*P*	0.992	0.052	0.041
				↓	↓
Field location (edge/centre)	1,13	*F*	24.01	21.47	5.68
		*P*	0.0003	0.0005	0.033
			↑	↑	↑
Insecticide treatment×Field location	1,13	*F*	<0.01	1.37	3.67
		*P*	0.945	0.263	0.078

Field identity was used as a random effect. Response variables were transformed (see statistical analyses). Mean and SE are shown in Fig. 4.

↑ = insecticide treated (yes) or edge is higher, ↓ = insecticide treated (yes) or edge is lower.

**Figure 5:**
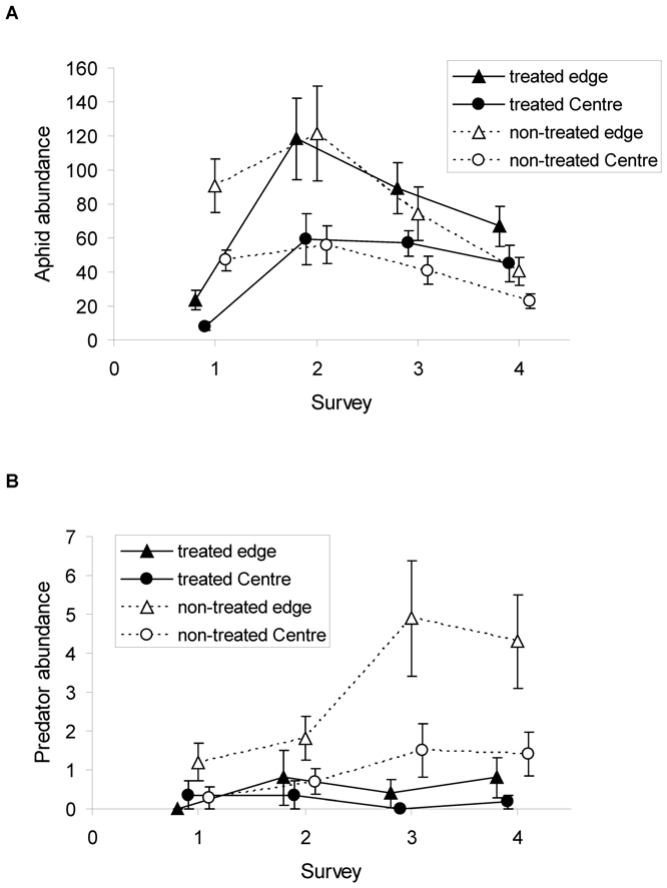
Temporal dynamics of (a) aphid abundance and (b) predator abundance in insecticide treated and non-treated conventional triticale field edges and centres (mean ±se). Statistics see Table 5.

To find the potential mechanism behind these patterns we analysed the temporal dynamics of aphids and their predators during the 4 surveys. Aphid abundance was significantly reduced after insecticide application only in the first survey, but afterwards rapidly increased. In the last survey there was even a trend for higher aphid abundances in sprayed compared to unsprayed conventional fields (Fig. 5a, Table 5). In a mixed effect model including time step 1 and time step 4 in one model (see statistical analyses), the interaction term between insecticide treatment×time step was highly significant (*F*_1,13_ = 20.98, *P*<0.001), providing evidence that the effect of insecticide treatment was not constant across the sampling dates, with lower aphid abundances in the treated fields at the first survey (first time step), but higher aphid abundances in the treated fields at the last survey. The generally low abundance of predators at the first two surveys was not affected by insecticide application. However the third and fourth surveys show significantly higher abundances for predators in non-treated fields, indicating a time delayed response to the insecticide treatment (Fig. 5b, Table 5). During the four surveys aphid abundances were consistently higher at field edges, while predator abundances were higher at field edges only at the last two surveys (Fig. 5, Table 5).

**Table 5:** Mixed effects model statistics for temporal changes in aphid abundance and predator abundance with the explanatory variables insecticide treatment, flied location and the interaction of insecticide treatment and field location.

	df		Aphid Survey 1	Aphid Survey 2	Aphid Survey 3	Aphid Survey 4
Insecticide treatment (yes/no)	1,13	*F*	22.40	<0.01	1.92	4.02
		*P*	0.0004	0.989	0.190	0.066
			↓			↑
Field location (edge/centre)	1,13	*F*	54.31	9.51	13.51	30.54
		*P*	<0.0001	0.009	0.003	0.0001
			↑	↑	↑	↑
Insecticide treatment×Field location	1,13	*F*	8.07	0.42	0.50	0.49
		*P*	0.014	0.528	0.492	0.493

	df		Predator Survey 1	Predator Survey 2	Predator Survey 3	Predator Survey 4
Insecticide treatment (yes/no)	1,13	*F*	1.41	1.38	6.80	7.25
		*P*	0.257	0.261	0.022	0.019
					↓	↓
Field location (edge/centre)	1,13	*F*	1.81	2.21	11.85	20.82
		*P*	0.202	0.161	0.004	0.0005
					↑	↑
Insecticide treatment×Field location	1,13	*F*	3.75	0.68	1.43	2.42
		*P*	0.075	0.424	0.253	0.144

Field identity was used as a random effect. Response variables were transformed (see statistical analyses). Mean and SE are shown in Fig. 5.

↑ = insecticide treated (yes) or edge is higher, ↓ = insecticide treated (yes) or edge is lower.

## Discussion

Our results show that organic farming increases biodiversity of vascular plants and pollinators, as well as vegetation cover and pollinator abundances. In addition the abundances of aphidophagous predators were enhanced, allowing a better top down control of aphids, which had clearly lower abundances in organic compared to conventional triticale fields. Insecticide spraying in conventional fields did decrease aphid abundances, but only for a short time period. After two weeks the insecticide effect was gone and at the end of the season aphid abundances were even higher in sprayed fields compared to not sprayed fields. In contrast, abundances of aphid-predators remained low in insecticide sprayed fields throughout the study period, but did increase their abundance in not sprayed fields.

We show that vascular plant species richness and vegetation cover are higher in organic compared to conventional fields, as similarly shown for other study systems [13], [15], [41], [42]. In general most functional species groups have higher species diversities and abundances in organic fields [6], [12]. Pollinator diversity and abundances in organic wheat fields in Germany were also enhanced [20], but not in organic tomato and watermelon fields in the USA [43]. In our organic triticale fields we found more species and higher abundances of pollinators, which can provide important ecosystem services for wild plants. These enhanced pollinator numbers might be also linked to the correlation between pollinator diversity and plant diversity in the triticale fields. Such correlations have been also reported for other study systems [20], [44].

We assume that pest control is enhanced in organic compared to conventional fields because of a higher predator-prey ratio, a free ecosystem service which needs further exploration [6], [45]. Cereal aphids are disastrous pests across the world, and their control can be time-consuming and cost intensive for farmers. The aphid species *R. padi* alone has the potential to decrease the yield of barley by 52% [46]. In our organic triticale fields the predator-prey ratio was almost 20 times higher compared to the conventional fields. However, this enhanced pest control was not only evident for organic vs. conventional fields, but also within conventional fields where we found higher predator-prey ratios in insecticide untreated fields compared to insecticide treated fields. This seems surprising, as the insecticide was sprayed to reduce cereal aphid abundances. However we show that this aphid reduction works only for a short time period. Afterwards aphid abundances increased rapidly, which is typical for aphid phenologies [26]. Most aphid predators are insects and could have been simultaneously poisoned after insecticide applications. The used insecticides, Karate®Zeon and Pirimor, were described on the one side as specific for sucking or chewing herbivores, or even as specific for aphids (Pirimor), but on the other side as harmful for several aphid predators like ladybirds, syrphids and green lacewings (Syngenta product information: http://www.syngenta.de/). More herbivore specific insecticides could essentially reduce negative effects of insecticide application on beneficial insects. Aphid predators usually occur in low densities at the beginning of the season and increase their abundances following the availability of their aphid prey [28], [47]. However in the sprayed conventional triticale fields the aphid predators did not increase their abundances, beside an increase in aphid abundances, during the study period. In contrast aphid predator abundance increased in the not sprayed conventional fields. Possible explanations for the low abundances of predators at the end of the study period in the sprayed fields are that (1) the insecticides were systemic and prevented development of aphid predators or that (2) colonisation of aphid predators was delayed due to low aphid abundances directly after the insecticide treatment. In a pan European study it was shown that the predation rate on aphids in cereal fields declined, when farmers increased the amount of applied insecticides [26].

Due to the low number of pollinators in conventional triticale fields, we could not test if pollinators showed similar negative responses to insecticide treatment. A recent study indicates that wild bee species are negatively affected by insecticide treatments in agricultural systems, at least when two insecticide treatments in the growing season were conducted [48]. However, the low densities of pollinators in conventional triticale fields without insecticide application suggest that enhanced diversity of pollinators in organic fields is mainly related to higher floral resource availability.

Apart from differences between triticale fields, we also showed that field edges within fields are more important for species diversity and ecosystem services than field centres. Field edges contained higher plant and pollinator diversity as well as higher predator abundances and higher predator-prey ratios. Previous studies also reported that field edges contain higher diversities of plants, spiders, beetles and pollinators [15], [49]. These edge effects might be caused by lower farming intensity at field edges or spillover from adjacent habitats [50]. Field edges therefore contribute essentially to species diversity and ecosystem services in cereal fields.

We conclude that organic farming contributes to the maintenance of biodiversity in agricultural systems. Organic farming also enhances species groups that provide ecosystem services with benefits for farmers due to better top-down control of pest species. The preventative insecticide application in conventional fields had significant direct costs in terms of material and labour with no long-term benefit for aphid control and negative effects on natural antagonists. As all triticale fields had relatively low aphid abundances we assume that the aphid abundances in cereals in our study year 2008 were below an economic injury level. We therefore conclude that the application of insecticides without a priori monitoring of aphid abundances and below critical thresholds increases direct management costs for farmers and indirect costs due to reduced ecosystem services like an effective biological pest control.

## Supporting Information

Material S1 APPENDIX A. Plant species recorded in organic and conventional fields. APPENDIX B. Pollinator species recorded in organic and conventional fields.
(DOC)
