# Supplementary material for: Decreased Functional Diversity and Biological Pest Control in Conventional Compared to Organic Crop Fields
Source: PLoS One. 2011 May 18;6(5):e19502. doi: 10.1371/journal.pone.0019502 (PMC3097189; doi:10.1371/journal.pone.0019502)
Supplement: Material S1 — APPENDIX A. Plant species recorded in organic and conventional fields. APPENDIX B. Pollinator species recorded in organic and conventional fields. (DOC) [file pone.0019502.s001.doc]

**Supplementary material**

**Appendix A.** Plant species recorded in organic and conventional fields.

| **Plant species** | | | | **organic** | **conventional** |
| --- | --- | --- | --- | --- | --- |
| *Acer platanoides* L. | | |  | x |  |
| *Achillea millefolium* L. | | |  | x | x |
| *Aethusa cynapium* L. | | |  | x | x |
| *Agrostis stolonifera* L. | | |  | x |  |
| *Alopecurus myosuroides* Huds. | | | | x | x |
| *Anagallis arvensis* L. | | |  | x | x |
| *Anchusa arvensis* (L.) M. Bieb. | | | | x |  |
| *Anthemis arvensis* L. | | |  | x |  |
| *Apera spica-venti* (L.) P. Beauv. | | | | x | x |
| *Aphanes arvensis* L. | | |  | x | x |
| *Arabidopsis thaliana* (L.) Heynh. | | | | x |  |
| *Arenaria serpyllifolia* L. | | | | x |  |
| *Atriplex patula* L. | | |  | x |  |
| *Avena fatua* L. | | |  | x | x |
| *Avenella flexuosa* L. Drejer | | | |  | x |
| *Brassica napus* L. | | |  | x |  |
| *Calystegia sepium* (L.) R. Br. | | | | x |  |
| *Capsella bursa-pastoris* (L.) Med. | | | | x | x |
| *Centaurea cyanus* L. | | |  | x | x |
| *C. jacea* L. | | |  | x |  |
| *Cerastium arvense* L. | | |  | x |  |
| *Chenopodium album* L. | | | | x | x |
| *Cirsium arvense* (L.) Scop. | | | | x | x |
| *C. oleraceum*  (L.) Scop. | | | | x |  |
| *Convolvulus arvensis* (L.) | | | | x | x |
| *Cynosurus cristatus* L. | | |  |  | x |
| *Daucus carota* L. | | |  |  | x |
| *Deschampsia flexuosa*  (L.) Trin. | | | | x |  |
| *Echinochloa crus-galli* (L.) Beauv. | | | | x | x |
| *Elymus repens* (L.) Gould | | | | x | x |
| *Epilobium montanum* L. | | | | x |  |
| *Equisetum arvense* L. | | |  | x | x |
| *E. palustre* L. | | |  |  | x |
| *Erysimum cheiranthoides* L. | | | | x |  |
| *Euphorbia cyparissias* L. | | | | x |  |
| *E. helioscopia* L. | | |  | x | x |
| *Fagopyrum esculentum* Moench | | | | x |  |
| *Festuca ovina* L. | | |  |  | x |
| *F. pratensis* Huds. | | |  |  | x |
| *Fumaria officinalis* L. | | |  | x | x |
| *Galeopsis ladanum* L. | | |  | x |  |
| *G. tetrahit* L. | | |  | x |  |
| *Galium aparine* L. | | |  | x | x |
| *Geranium dissectum* L. | | | | x | x |
| *G. pratense* L. | | |  | x |  |
| *G. robertianum*  L. | | |  | x | x |
| *Gnaphalium uliginosum* L. | | | | x | x |
| *Hypochaeris glabra* L. | | |  | x |  |
| *Kickxia elatine* (L.) Dumort. | | | | x |  |
| *K. spuria* (L.) Dumort. | | |  | x |  |
| *Lamium amplexicaule* L. | | | | x |  |
| *L. maculatum* L. | | |  | x |  |
| *L. purpureum* L. | | |  | x | x |
| *Lapsana communis* L. | | |  | x |  |
| *Lathyrus pratensis* L. | | |  | x | x |
| *Leucanthemum vulgare* L. | | | | x |  |
| *Linaria minor* L. Desf. | | |  | x |  |
| *Lolium multiflorum* Lam. | | | | x |  |
| *Lotus corniculatus* L. | | |  | x |  |
| *Matricaria discoidea* DC. | | | | x |  |
| *M. recutita* L. | | |  | x | x |
| *Medicago lupulina* L. | | |  | x | x |
| *Mentha arvensis* L. | | |  | x |  |
| *Moehringia trinervia* (L.) Clairv. | | | | x |  |
| *Myosotis arvensis* (L.) Hill | | | | x | x |
| *Myosurus minimus* L. | | |  | x |  |
| *Oxalis stricta* L. | | |  | x |  |
| *Papaver rhoeas* L. | | |  | x |  |
| *Phleum pratense* L. | | |  | x | x |
| *Phragmites australis*  Trin. ex Steud. | | | | x |  |
| *Plantago lanceolata* L. | | |  | x | x |
| *P. major*  L. | | |  | x |  |
| *Poa annua* L. | | |  | x | x |
| *P. compressa* L. | | |  | x |  |
| *P. nemoralis* L. | | |  | x |  |
| *P. trivialis*  L. | | |  | x |  |
| *Polygonum aviculare* L | | . | | x | x |
| *P. convolvulus* L. | | |  | x | x |
| *P. lapathifolium* L: | | |  | x | x |
| *P. persicaria* L. | | |  | x | x |
| *Potentilla reptans* L. | | |  | x | x |
| *Prunus spinosa* L. | | |  | x |  |
| *Quercus robur* L. | | |  |  | x |
| *Ranunculus arvensis* L. | | |  | x |  |
| *R. repens* L. | | |  | x | x |
| *Raphanus raphanistrum* L. | | | | x |  |
| *Rumex acetosa* L. | | |  | x |  |
| *R. acetosella* L. | | |  | x |  |
| *R. crispus* L. | | |  | x | x |
| *Sagina procumbens* L. | | |  | x |  |
| *Sambucus nigra* L. | | |  | x | x |
| *Scleranthus annuus* L. | | |  | x |  |
| *Setaria viridis* (L.) Beauv. | | | | x | x |
| *Sherardia arvensis* L. | | |  | x |  |
| *Sinapis arvensis* L. |  | | | x |  |
| *Sonchus arvensis* L. | | |  | x |  |
| *S. asper* (L.) Hill | | |  | x |  |
| *S. oleraceus* L. | | |  | x |  |
| *Spergula arvensis* L. | | |  | x |  |
| *Stachys palustris* L. | | |  | x |  |
| *Stellaria media* (L.) Vill. | | | | x | x |
| *Tanacetum vulgare* L. | | |  | x |  |
| *Taraxacum officinale* Weber ex Wiggers | | | | x | x |
| *Thlaspi arvense* L. | | |  | x | x |
| *Trifolium arvense* L. | | |  | x |  |
| *T. campestre* Schreb. | | |  | x |  |
| *T. dubium* Sibth. | | |  | x |  |
| *T. pratense* L. | | |  | x |  |
| *T. repens* L. | | |  | x | x |
| *Triticum aestivum* L. | | |  | x |  |
| *Tussilago farfara*  L. | | |  | x |  |
| *Valerianella locusta* (L.) Laterr. | | | | x | x |
| *Veronica arvensis* L. | | |  | x |  |
| *V. hederifolia* L. | | |  | x |  |
| *V. persica* Poir. | | |  | x | x |
| *Vicia angustifolia* L. | | |  | x |  |
| *V. cracca* L. | | |  | x | x |
| *V. hirsuta* (L.) Gray | | |  | x | x |
| *V. sativa* L. | | |  | x |  |
| *V. sepium* L. | | |  |  | x |
| *V. villosa* Roth | | |  | x |  |
| *Viola arvensis* Murray | | |  | x | x |

**Appendix B.** Pollinator species recorded in organic and conventional fields.

| **Pollinator species** |  | **organic** | **conventional** |
| --- | --- | --- | --- |
| *Aphantopus hyperantus* L*.* | | x |  |
| *Apis mellifera* L. | | x | x |
| *Argynnis paphia* L. | | x |  |
| *Bombus hortorum* L. | | x |  |
| *B. lapidarius* L. | | x | x |
| *B. pascuorum* SCOPOLI | | x | x |
| *B. pratorum* L. | | x |  |
| *B. terrestris* L. | | x | x |
| *Cheilosia chloris* MEIGEN | | x |  |
| *Epistrophe melanostoma* ZETTERSTEDT | | x |  |
| *Episyrphus balteatus* De Geer | | x | x |
| *Eristalis pertinax* SCOPOLI | | x |  |
| *E. tenax* L. |  | x |  |
| *Ferdinandea cuprea* SCOPOLI | | x |  |
| *Gonepteryx rhamni* L. | | x |  |
| *Helophilus pendulus* L. | | x |  |
| *Maniola jurtina* L. | | x |  |
| *Melanostoma scalare* FABRICIUS | | x |  |
| *Nymphalis io* L. | | x |  |
| *Parasyrphus annulatus* ZETTERSTEDT | | x |  |
| *Pieris brassicae* L. | | x |  |
| *Scaeva pyrastri* L. | | x |  |
| *Sphaerophoria scripta* L. | | x |  |
| *Syritta pipiens* L. | | x |  |
| *Syrphus ribesii* L. | | x |  |
| *S. torvus* OSTEN-SACKEN | | x |  |
| *S. vitripennis* MEIGEN | | x |  |
| *Thymelicus lineola* OCHSENHEIMER | | x |  |
| *T. sylvestris* PODA | | x |  |
| *Vanessa cardui* L. | | x |  |
| *Volucella pellucens* L. |  | x |  |
